# AGING SUSTAINABLY: A PILOT STUDY OF OLDER MIDWESTERN ADULT PERSPECTIVES ON ENVIRONMENTAL BEHAVIOR

**DOI:** 10.1093/geroni/igad104.3174

**Published:** 2023-12-21

**Authors:** Megan Pedersen, Justin Walden, Melissa O’Connor

**Affiliations:** North Dakota State University, Fargo, North Dakota, United States; North Dakota State University, Fargo, North Dakota, United States; North Dakota State University, Fargo, North Dakota, United States

## Abstract

Given their life experience and significant political power, the perspectives of older adults on environmental issues are crucial to addressing the climate crisis. Although unsupported by research, the general public tends to believe that older adults do not care about climate change. In seeking to overturn this belief, a qualitative responsive interview study was conducted. With a sample of 5 adults between 50 and 70 from North Dakota and Minnesota, this study is small, but can serve as a pilot study for more intensive research. Interview questions focused on older adults’ attitudes, personal behavior, and motivations for engaging (or not) in sustainable behavior. Although the sample size was small, findings showed that most older adults understand the need for sustainability and try to care for the climate in their own ways. Religion, financial incentive, and generativity can be strong motivators for sustainable behavior, but misinformation and distrust can act as de-motivators. Investigating older adults’ motivation to engage in sustainable behavior is a necessary step to involving more older adults in environmental advocacy. These interviews demonstrate that many older adults are willing to act on behalf of our shared planet, and future research may uncover more motivations and bring us closer to that goal.

